# Description of Main Predictors for Taking Sick Leave Associated with Work-Related Eye Injuries in Spain

**DOI:** 10.3390/ijerph18105157

**Published:** 2021-05-13

**Authors:** Sergio Martín-Prieto, Cristina Alvarez-Peregrina, Israel Thuissard-Vassallo, Carlos Catalina-Romero, Eva Calvo-Bonacho, César Villa-Collar, Miguel Ángel Sánchez-Tena

**Affiliations:** 1 Faculty of Biomedical and Health Sciences, Universidad Europea de Madrid, 28670 Madrid, Spain; martin.prieto.sergio@gmail.com (S.M.-P.); israeljohn.thuissard@universidadeuropea.es (I.T.-V.); villacollarc@gmail.com (C.V.-C.); masancheztena@gmail.com (M.Á.S.-T.); 2 Ibermutua (Mutual Collaborator of Social Security nº 274), 28043 Madrid, Spain; carloscatalina@ibermutua.es (C.C.-R.); delgatest@yahoo.es (E.C.-B.)

**Keywords:** work-related eye injuries, sick leave, occupational injuries, injury epidemiology, ocular injuries, labor absence costs

## Abstract

Recent studies in Spain have shown that males, younger workers, and people involved in manual jobs had the highest risk of suffering a work-related eye injury (WREI). This study aims to assess the predictors of sick leave associated with WREI and to compare them with risk factors of initial injury. A retrospective and descriptive study of WREI that causes sick leave of one or more days among workers from an insurance labor mutual company in Spain was conducted over a period from 2008 to 2018. The variables of the study were sex, age, occupation, and type of injury. A total of 9352 (18.6% of 50,265 WREI) cases and 113,395 total days of sick leave were observed, with an estimated EUR 4,994,009.59 of associated labor cost. The main predictors of sick leave related to WREI were found to be female (highest incidence; 25.9 (95% CI (24.8–27.1))), >55 years of age (highest incidence; 20.5 (95% CI (19.3–21.7))), not working in the industry (lowest incidence; 13.8 (95% CI (13.3–14.2))), and not suffering “other disorders of conjunctiva” (lowest incidence; 5.7 (95% CI (4.7–6.8))). The consequences associated with WREI are worse for female and older workers, despite the main risk of suffering WREI being observed in males and younger workers.

## 1. Introduction

The European Statistic on Accidents at Work (ESAW) defines sick leave depending on the number of days of absence from work due to a work-related injury [1]. There are few studies about work-related eye injuries (WREI) in Spain, and all of them included very few subjects. Thus, in 1988, Mencía-Gutierrez et al. reviewed 77 patients over five years that came to the hospital with perforating ocular wounds caused by working accidents. The industry sector was the one with the most cases (61%), followed by the sectors of agriculture and services (26% and 13%, respectively) [2]. In 2005, Olmos-Zapata et al. published a retrospective study between 1996 and 2001. They followed 25 patients requiring hospitalization and surgery for serious ocular traumatism due to a foreign intraocular body. Of those accidents, 72% occurred at work, and agriculture was the sector with the highest number [3]. In 2008, Serrano et al. found that 26% of the eye injuries attended to in emergency departments were related to work accidents. Of these work accidents, 61% were due to a foreign body, 17% were due to trauma, 17% were due to burns, and 2% were due to infections [4]. In 2010, Larque-Daza et al. analyzed 94 eyes with open globe trauma evaluated between 1999 and 2007 in a primary referral hospital from the southeast of Spain. Of the globe traumas, 56% were caused at work, and the types of injury included ruptures, intraocular foreign bodies, perforating injury, penetrating injury, and mixed injury [5]. A recent study from Guzman-Almagro et al. analyzed data from 104 patients that were attended to in a hospital for traumatic open globe injuries between 2011 and 2015. They concluded that more than half of the cases were work-related accidents, and their main mechanism was penetrating trauma or a foreign body [6]. 

Regarding the evaluation of the relationship of WREI with the total days of absence from work, Gómez Villa P et al. analyzed two populations in Mallorca (Spain) from 2005 to 2006. They found 392 WREI that provoked 1939 days of absence from work [7]. In addition, Moreno-Arrones et al. studied 4762 occupational eye injuries recorded during 2014 and 2015. Of the WREI, 43% were due to a foreign body in the eye, and chemical burns were found to be the second highest causes of WREI. They found that 75% of the chemical burns resulted in less than seven days of absence from work and that 20% of them were under 30 days of absence [8]. However, it would be interesting to analyze the predictive factors for these absences caused by WREI.

According to Ministerio de Trabajo, Migraciones y Seguridad Social (government of Spain), the evolution in the incidence of sick leave in Spain from 2008 to 2018 shows a decrease between 2008 and 2012, with the lowest value occurring in 2012–2013, and an upward trend starting from 2013 [9]. The maximum incidence of sick leave was registered in 2018 (617,488 accidents resulted in sick leave—46% of the accidents occurring at work), with an incidence of 3408.70 cases per 100,000 workers [9]. Three percent of the total number of sick leave were due to an injury of an ocular structure (17,579), with eyes being the most affected structures of the head [10].

The results of a previous study carried out by Martín-Prieto S. et al. in 2020 showed a WREI incidence in Spain of 429.75 per 100,000 workers from 2008 to 2018 [11]. The study also concluded that the risk of suffering a WREI was higher for males, younger and less experienced workers, and those who work with manual tasks, which agrees with previous studies [12,13,14,15,16]. In the analysis of other countries such as the USA, this kind of injury resulted in a median of two days of absence from work [17], and the total number of ocular injuries (either related or not related to work) incurred estimated costs of AUD 155 million and USD 200 million in Australia and the USA, respectively [18,19].

Analyzing the costs and days of absence associated with work accidents or work-related illness is a regular practice in some injuries such as cardiovascular diseases [20] or anxiety disorders [21]. A study of the different variables that cause an absence from work to occur can help to predict them.

Therefore, this study aims to analyze the risk factors for taking sick leave due to a work-related eye injury as well as the cost associated with these absences.

## 2. Materials and Methods

A descriptive and retrospective study was carried out using the database of Ibermutua, a mutual insurance company in Spain. This mutual insurance company evaluates the injuries suffered at work of their clients, collaborating with National Social Security to administer statutory sick pay. 

Each WREI that became a sick leave from 1 January 2008 to 31 December 2018 was analyzed. A WREI was considered any injury that occurred at work or on the way to work and that was classified in a medical examination into group VII (diseases of the eye and adnexa), between the codes H00 and H59 of the International Statistical Classification of Diseases and Related Health Problems 10th Revision (ICD-10), World Health Organization [22]. One or more days of absence from work were considered sick leave.

The main variables studied were gender, age, occupational sector, and the kind of eye injury caused by an accident at work. Any kind of injury that entailed less than 1% of the cases were classified as “other injuries”, according to the ICD-10 classification.

The Average Incidence (AI) of sick leave per 1000 WREI for each variable was calculated throughout the study period. For the study of the evolution of sick leave over the study period, we used a negative binomial regression to calculate an Average Annual Percentage Change (2008–2018 (AAPC [%])). Time trends for the study period were also reported as a Cumulative Percentage Change (CPC).

A quantitative statistical analysis (mode, median, and mean) of all of the variables was carried out and a Wald univariate logistic regression was used to reduce the number of potential predictors of absence from work. The factor with the lowest AI was taken as a reference. The correlation between different predictors and the comparison between the percentage of injury incidence, with and without sick leave, was analyzed using Pearson’s Chi-square test.

To calculate an employee’s salary cost, 75% of their daily salary (quotation based on the month before the sick leave, divided by 30 days) was multiplied by the number of days absent from work. 

The data analysis was performed with IBM SPSS statistics version 21.0 (IBM Corp, Armonk, NY, USA), and *p* < 0.05 was considered significant for statistical analyses. 

The research described herein adhered to the tenets of the Declaration of Helsinki and was approved by the ethics investigation committee of Universidad Europea de Madrid (CEI-UE). All medical records were anonymous; only statistical information was provided by Ibermutua for research purposes.

## 3. Results

The total number of people insured by Ibermutua between 2008 and 2018 was 11,696,259, and a total of 50,265 WREI occurred. These injuries resulted in one or more days of absence from work for 18.6% of the cases (9352). The average was 12.13 (±49.70) days of absence from work, and the total of days absent was 113,395. The mode was two days of absence from work (20.7%), with a median of 4 days (P25 = 2; P75 = 7). The mean age of the sample was 39.16 (±10.72) years old. Most of the absences occurred in men (7858; 85.0%), in the age group between 35–44 years (2929; 31.5%), in those who worked in the service sector (2963; 37.6%), and were most commonly caused by keratitis (6995; 74.8%).

### 3.1. Incidence of Labor Absence Per 1000 in Each Variable

The descriptive analysis and the incidence of taking sick leave for each 1000 WREI in all variables from 2008 to 2018 showed that the lowest AI was found in men (17.7 (95% CI 17.3–18.0)), in the age group of 16–24 (17.3 (95% CI 16.1–18.4)), in the industry sector (13.8 (95% CI 13.3–14.2)), and in those who suffered “other disorders of conjunctiva” (5.7 (95% CI (4.7–6.8)). Table S1 (Supplementary Material) shows the details of this descriptive analysis. 

The evolution of incidence (AI) during the study period showed two different stages: 2008–2013 and 2013–2018. From 2008 to 2013, there was hardly a 1.1% growth in the AAPC (95% CI −3.4% to 5.7%) and 3.0% growth in the CPC. However, between 2013 and 2018, there was a significant increase of 21.4% (95% CI 3.0 to 43.0) in the AAPC (%). The period-round analysis shows a significant increase (*p* < 0.05) in the AAPC (%) (5.7% (95% CI 2.7 to 8.8)) and an increase in the CPC of 94.3% of incidence when comparing 2008 with 2018 (Table S2, Supplementary data).

### 3.2. Sick Leave Predictors

A multivariate analysis showed that the main predictors for taking sick leave due to a WREI were being a woman (OR 1.63 (95% CI 1.51–1.77), being more than 55 years old (OR 1.16 (95% CI 1.03–1.31)), not work in the industry sector, and suffering an injury other than “other disorders of conjunctiva” (Table 1). Of all sectors, the highest risk was for those working in agriculture (OR 2.68 (95% CI (2.38–3.01)). The injuries with the highest risk of taking sick leave were those that affect the cornea (“keratitis”; OR 6.14 (95% CI [4.95–7.62]) and “other disorders of cornea”; OR 6462 (95% CI [4.89–8.55])).

### 3.3. WREI and Labor Absence Relationship

Table 2 shows the relationship between the percentage of WREI incidence with and without sick leave for each variable. The sex analysis shows a significant decrease in the percentage of incidences that cause sick leave for men (*p* < 0.05) compared to an increase in the incidences of sick leave for women (*p* < 0.05).

There was a decrease in the incidence for the age groups of 16–24 and 25–34 (*p* < 0.05). However, there was an increase in the 45–54 and >55 age groups (*p* < 0.05). There were no differences in the 35–44-year-old group. Regarding work sectors, there was a significant decrease in the industry (*p* < 0.05) and an increase in agriculture and services (*p* < 0.05). There were no differences in the construction sector.

Analyzing the kind of injuries caused by WREI, significant differences were found in all of them (*p* < 0.05). In “keratitis”, “other injuries”, “other disorders of the cornea”, and “disorders of the globe”, the percentage of incidences in cases with a sick leave increased. On the other hand, there was a decrease in the percentage of incidences in cases that took sick leave related to “conjunctivitis”, “visual disturbances”, “other disorders of the eye and adnexa”, and “other disorders of conjunctiva”.

### 3.4. Total Sick Leave Costs Associated with WREI

The cost analysis of 7818 cases registered as sick leave was carried out. The average daily cost was EUR 38.37 ± EUR 41.40 per employee, with a median of EUR 35.56 (P25: EUR 29.72; P75: EUR 44.27). Considering the days of absence from work of each employee, the average daily cost of each one was EUR 38.87 ± 41.50. With this data, the estimated total cost in all employees’ salaries throughout the period was EUR 4,994,009.59. 

Table 3 shows the salary cost estimation depending on different variables.

The highest daily average of salary cost was observed in men (39.20 ± 44.62), in those over 55 years old (42.37 ± 16.62), those working in the services sector (40.82 ± 69.94), and those who suffered “conjunctivitis” (42.42 ± 16.70). The estimated total cost was highest in all of the groups with more cases: men (4,221,496.41), the age group of 35–44 years (1,475,078.70), those who have suffered “keratitis” (2,349,016.02), and those working in the services sector (1,742,186.90).

## 4. Discussion

The scientific literature about the risk factors that explain sick leave due to any injury shows that those workers that work manual jobs used to suffer more sick leave [23]. Socioeconomic factors are also relevant, and workers with lower education levels, lower incomes, and poorly paid jobs have a higher risk of needing a sick leave [24,25]. The analysis of anxiety disorders follows the same pattern [21].

In the analysis of eye injuries at work, the risk factors for suffering a WREI previously reported by other studies [11,12,13,14,15,16,26,27] were being a man, having less experience, and working in manual jobs. However, the predictive factors for ahigher incidence of taking sick leave due to a WREI do not follow this pattern. Women and workers over 55 were the groups with the highest risks of taking sick leave. These results suggest that, although younger workers have a higher risk of suffering a WREI, the consequences of these are worse for older workers, possibly related to the different physical conditions between the two age groups. Women have a higher risk of taking sick leave, as Melchior et al. and Feeney et al. also reported in their analyses of different injuries related to work [23,28], without finding any specific reason for this higher risk. It might be related to the jobs that women usually perform, but there is no evidence of a direct relationship. In this study, there was an increase in the incidence of sick leave in the services sector, where the percentage of women at work used to be higher.

The average number of days of absence from work found in our study was higher than the one found in Taiwan (12.13 ± 49.70 vs. 8.3 ± 7.5 days) [29] and in Australia (2.0 ± 2.3 days) [30]. It may be due to the lower number of cases and periods studied in both studies of Taiwan and Australia. However, the median number of days of absence from work was similar to the median found in other studies in the USA [17]. The median is, in this case, a better statistic for comparison between studies, as it decreases the relevance of the dispersion of data that exists in broad samples.

The WREI incidence for 100,000 insurance employees decreased over the period, according to the previous work of Martín-Prieto et al. [11]. However, the incidence for the number of days of absence from work related to WREI increased from the beginning to the end of the period due to the increase observed starting from 2013. This difference between incidences of WREI and sick leave related to WREI could be due to an increase in the prevention of labor risk policies that governments, companies, and insurance companies have taken. The significant increase from 2013 could be due to the implementation of a new labor law in Spain in 2012 that seems to have increased temporary workers [31]. This change in the law could even cause not many employees to notify the ministry about minor injuries related to work; therefore, probably only the most serious injuries were reported.

According to our previous study, working in the industry sector is supposed to have a 7.75 times higher risk of suffering a WREI [11]. However, in the present study, it was the sector with a lower risk of taking sick leave. That means that accidents from the industry sector are less serious than accidents in other sectors. This might be due to the better protection measures takes in the industry sector compared to other sectors. In a previous analysis, lesions affecting the cornea and conjunctiva were the most common type of injury in a WREI [32]. However, those WREIs that affect the conjunctiva were the ones that had the lowest risk of requiring sick leave. This indicates that, although eyes are easily affected structures, injuries at work do not tend to be serious. On the other hand, WREIs that affects the cornea and those that generate “visual disturbances” are the ones that cause the most harm to employees. Protecting the ocular surface by wearing protective spectacles, taking ergonomic measures that avoid visual fatigue, and increasing eye hydration when working with screens are simple and effective measures to prevent sick leave related to WREI.

The estimated labor costs of sick leave from a WREI declared by the insurance company that we analyzed over the 11 years of study are high (EUR 4,994,009.59), although it is much lower than those that caused cardiovascular injury in Spain (EUR 5,801,464.18 a year) [15]. It is important to highlight that this is only a government cost and that it only includes the salary cost that is covered by National Social Security (NSS). In Spain, NSS covers 75% of an employee’s NSS quotation base. This statistic underestimates the actual cost since it does not include additional costs faced by companies. Companies should pay 33% of the quotation base of the employee during his/her sick leave. Moreover, most companies in Spain also compliment the NSS payment such that the employee receives up to 100% of his/her usual salary. Therefore, considering that 11 years of sick leave due to WREI have resulted in a cost of almost EUR 5 million and the cost that companies used to hire new people to substitute the employees under sick leave, the estimated cost for the companies would have been more than EUR 10 million (100% of the cost of the substitute, 33% of the social security cost of the replaced employee, plus the compliment up to 100% that the company usually pays). Other costs that are not included in this estimation are medical care costs, pharmacy costs, and other costs due to the training that the substitute needs to perform his/her new job (learning curve).

We could not find previous studies to analyze the salary costs from sick leave due to eye injuries across Europe, although Quesada et al. estimated some of the costs of ocular chemical burns in 2004 and 2005 in Spain. Our study could serve as a starting point to implement effective prevention policies to make savings in these costs. Eye structures are simple to protect, and prevention policies could avoid between 60–66% of WREI, generating savings as shown in the study made in Australia with a saving estimation of about $37 million [33]. The total estimated cost for each variable agrees with those variables that have the highest number of cases (men, age group between 35–44 years, workers in the services sector, and those suffering from “keratitis”) despite the differences between the average salary and the days of absence from work within the different groups. Therefore, some easy to implement measures such as the use of protection glasses, artificial tears, or some ergonomic measures to protect eyes could generate important savings.

This is the study with the highest number of cases over the longest period observed in Europe, and it is also the first that evaluates the economic consequences of sick leave related to WREI. However, it has also limitations, such as the difficulty of comparison with similar studies or the lack of salary data from some employees (we analyzed 7818 from 9352 WREI cases due to incomplete records). The analysis carried out does not give information about the reasons why women have a higher risk of taking sick leave due to a WREI. An analysis of only sick leave days is not enough to compare the relevance of the injury between men and women. It would be interesting in future studies to carry out a more thorough study in a small group of WREI cases to know which injuries provoke sick leave, which task the injured employee was doing, how the injury occurred, if he/she was wearing ocular protection, and even his/her education level. This deeper analysis would allow us to know in detail the associated costs of both the salary paid during sick leave and the medical costs of diagnosis and treatment.

Knowing the predictor factors of taking sick leave related to WREI is very important to plan effective prevention policies that help to protect employees’ eye structures and to avoid the sick leave-associated costs.

## 5. Conclusions

Being a woman, being more than 55 years old, not working in the industry sector, and suffering an injury in an ocular structure different from conjunctiva are the predictors for taking sick leave related to WREI.

Although risk factors for suffering a WREI are different from those that provoke a sick leave (being male, having little work experience, and working in the industry sector), these results suggest that their consequences are worse for women and older workers.

The direct salary costs of sick leave related to WREI come to an average of EUR 499,400.96 per year. These costs could decrease through the implementation of protection policies. The most effective ones would be those with an emphasis on the groups with the highest estimated cost: men, age 35–44 years, working in the services sector, and suffering from “keratitis”.

## Figures and Tables

**Table 1:** Predictors of sick leave in workers who suffered a work-related eye injury.

Variable	OR ^1^ (95% CI)	*p*-Value
**Sex**		
Male	REF.	
Female	1.63 (1.51–1.77)	<0.001
**Age**		
16–24	REF	
24–34	1.02 (0.92–1.13)	0.74
35–44	1.04 (0.94–1.15)	0.48
45–54	1.08 (0.97–1.19)	0.18
>55	1.16 (1.03–1.31)	<0.05
**Occupation**		
Agriculture	2.68 (2.38–3.01)	<0.01
Industry	REF.	
Construction	1.26 (1.18–1.35)	<0.01
Services	1.69 (1.59–1.80)	<0.01
**Eye injuries**		
H16	6.14 (4.95–7.62)	<0.01
H10	1.32 (1.06–1.65)	<0.05
Other injuries	4.93 (3.86–6.29)	<0.01
H18	6.46 (4.87–8.55)	<0.01
H44	5.24 (3.95–6.95)	<0.01
H53	20.9 (1.57–2.79)	<0.01
H57	1.90 (1.39–2.59)	<0.01
H11	REF.	

^1^ Odds ratios; H16, keratitis; H10, conjunctivitis; H18, other disorders of cornea; H44, disorders of the globe; H53, visual disturbances; H57, other disorders of the eye and adnexa; and H11, other disorders of conjunctiva.

**Table 2:** Statistical relationship between WREI with and without sick leave for each variable and the total percentage of sick leave over total WREI.

Variable	N WREI without Sick Leave and Percentage of Incidence (%)	N WREI with Sick Leave and Percentage of Incidence (%)	*p*-Value	Percentage of Sick Leave Over Total WREI (%)
**Sex**				
Male	36,587 (90.2)	7858 (85.0)	<0.001	17.68
Female	3961 (9.8)	1388 (15.0)	<0.001	25.95
**Age**				
16–24	3631 (8.9)	757 (8.1)	<0.05	17.25
25–34	12,298 (30.2)	2683 (28.8)	<0.05	17.91
35–44	13,063 (32.1)	2929 (31.5)	0.28	18.32
45–54	8247 (20.2)	2031 (21.8)	<0.001	19.76
>55	3489 (8.6)	901 (9.7)	<0.001	20.52
**Occupation**				
Agriculture	1101 (3.0)	523 (6.6)	<0.001	32.20
Industry	16,300 (44.7)	2599 (33.0)	<0.001	13.75
Construction	8657 (23.7)	1798 (22.8)	0.082	17.20
Services	10,431 (28.6)	2963 (37.6)	<0.001	22.12
**Eye injuries**				
H16	19,679 (48.1)	6995 (74.8)	<0.001	26.22
H10	14,754 (36.1)	1152 (12.3)	<0.001	7.24
Other injuries	1508 (3.7)	461 (4.9)	<0.001	23.41
H18	521 (1.3)	208 (2.2)	<0.001	25.24
H44	642 (1.6)	184 (2.0)	<0.01	22.27
H53	1130 (2.8)	133 (1.4)	<0.001	10.53
H57	916 (2.2)	112 (1.2)	<0.001	10.89
H11	1763 (4.3)	107 (1.1)	<0.001	5.72

H16, keratitis; H10, conjunctivitis; H18, other disorders of cornea; H44, disorders of the globe; H53, visual disturbances; H57, other disorders of the eye and adnexa; and H11, other disorders of conjunctiva.

**Table 3:** Total labor costs estimated by each variable.

Variable	N WREI with Sick Leave in Each Variable	Total Workers Considered	Media (SD) Days of Absence from Work Per Worker	Media (SD) Days of Absence from Work Cost (€)	Estimated Sick Leave Cost (€)
**Sex**					
Male	7858	6543	13.71 ± 55.36	39.20 ± 44.62	4,221,496.41
Female	1388	1186	14.28 ± 48.62	37.64 ± 18.72	746,264.99
**Age**					
16–24	757	617	7.74 ± 21.51	30.88 ± 10.85	180,951.50
25–34	2683	2257	11.38 ± 47.36	37.99 ± 73.43	1,160,308.10
35–44	2929	2433	12.74 ± 49.19	39.54 ± 14.64	1,475,078.70
45–54	2031	1700	17.90± 73.54	40.51 ± 15.87	1,472,571.97
>55	901	770	19,83 ± 55.46	42.37 ± 16.38	756,907.27
**Occupation**					
Agriculture	523	474	14.50 ± 51.185	31.50 ± 11.20	238,931.76
Industry	2599	1977	11.53 ± 56.49	40.77 ± 13.50	1,222,276.98
Construction	1798	1575	12.60 ± 55.67	37.79 ± 10.84	855,840.92
Services	2963	2647	14.40 ± 49.88	40.82 ± 69.94	1,742,186.90
**Eye injuries**					
H16	6995	5858	8.74 ± 41.02	38.43 ± 46.99	2,349,016.02
H10	1152	903	8.56 ± 22.09	42.42 ± 16.70	398,857.17
Other injuries	461	424	75.15 ± 124.85	41.45 ± 15.96	1,436,151.51
H18	208	177	18.41 ± 67.71	40.22 ± 16.79	154,051.33
H44	184	157	43.95 ± 110.35	39.13 ± 13.14	316,422.91
H53	133	110	27.11 ± 82.64	39.98 ± 15.89	144,132.68
H57	112	97	22.36 ± 66.96	37.64 ± 17.15	94,257.63
H11	107	92	14.32 ± 43.41	36.33 ± 16.37	55,655.30

H16, keratitis; H10, conjunctivitis; H18, other disorders of cornea; H44, disorders of the globe; H53, visual disturbances; H57, other disorders of the eye and adnexa; and H11, other disorders of conjunctiva.

## Data Availability

Ibermutua (Mutual collaborator of social security nº 274), Madrid 28043, Spain.

## Supplementary Materials

The following are available online at https://www.mdpi.com/article/10.3390/ijerph18105157/s1, Table S1: Demographic description of the total number of WREI and sick leaves over a total of 50,265 WREI; Table S2: Average incidence of sick leaves per 1000 WREI, accumulative average annual percent change, and cumulative percentage change in the incidence of all sick leaves over the study period (2008–2018).
