# Supplementary material for: Description of Main Predictors for Taking Sick Leave Associated with Work-Related Eye Injuries in Spain
Source: Int J Environ Res Public Health. 2021 May 13;18(10):5157. doi: 10.3390/ijerph18105157 (PMC8152250; doi:10.3390/ijerph18105157)
Supplement: Supplementary file 1 [file ijerph-18-05157-s001.zip › ijerph-1191483-supplementary.pdf]

Table S1: Demographic description of the total number of WREI and sick leaves over a total of 50 265 WREI. Average Incidence and Median duration in days of absence from work over 9 352 cases of labor absence are shown.

Variable	WREI[1] (%)	N days of absence from work (%)	AI[2] of days of absence from work per 1,000 WREI (95% CI)	Median of days of absence from work (95% CI)
**Sex**				
**Male**	44,445(89.3)	7,858 (85.0)	17.7 (17.3-18.0)	4.0 (1.5-6.5)
**Female**	5,349 (10.7)	1,388 (15.0)	25.9 (24.8-27.1)	5.0 (2.0-8.0)
**Age**				
**16-24**	4,388 (8.8)	757 (8.1)	17.3 (16.1-18.4)	4.0 (2.0-6.0)
**25-34**	14,981 (29.9)	2,683 (28.8)	17.9 (17.3-18.5)	4.0 (1.5-6.5)
**35-44**	15,992 (32.0)	2,929 (31.5)	18.3 (17.7-18.9)	4.0 (1.5-6.5)
**45-54**	10,278 (20.5)	2,031 (21.8)	19.8 (19.0-20.5)	4.0 (1.5-6.5)
**> 55**	4,390 (8.8)	901(9.7)	20.5 (19.3-21.7)	6.0 (2.0-10.0)
**Occupation**				
**Agriculture**	1,624 (3.7)	523 (6.6)	32.2 (29.9-34.5)	5.0 (2.5-7.5)
**Industry**	18,899 (42.6)	2,599 (33.0)	13.8 (13.3-14.2)	4.0 (2.0-6.0)
**Construction**	10,455 (23.6)	1,798 (22.8)	17.2 (16.5-17.9)	4.0 (1.5-6.5)
**Services**	13,394 (30.2)	2,963 (37.6)	22.1 (21.4-22.8)	4.0 (1.5-6.5)
**Eye injuries**				
**H16 Keratitis**	2, 674 (53.1)	6,995 (74.8)	26.2 (25.7-26.8)	4.0 (1.5-6.5)
**H10 Conjunctivitis**	15,906 (31.6)	1,152 (12.3)	7.2 (6.8-7.6)	4.0 (1.5-6.5)
**Other injuries***	1,969 (3.9)	461 (4.9)	23.4 (21.5-25.3)	18.0 (0.0-50.3)
**H18 Other disorders of cornea**	729 (1.5)	208 (2.2)	28.5 (25.3-31.8)	4.0 (1.5-6.5)
**H44 Disorders of Globe**	826 (1.6)	184 (2.0)	22.3 (19.4-25.1)	4.0 (0.0-11.5)
**H53 Visual disturbances**	1,263 (2.5)	133 (1.4)	10.5 (8.8-12.2)	4.0 (1.5-6.5)
**H57 Other disorders of eye and anexa**	1,028 (2.0)	112 (1.2)	10.9 (9.0-12.8)	5.0 (1.8-8.8)
**H11 Other disorders of conjunctiva**	1,870 (3.7)	107 (1.1)	5.7 (4.7-6.8)	5.0 (2.0-8.0)

1 Work-related eye injuries; 2 Average incidence; * Other WREIs that do not belong to the six main codes with International Classification of Diseases (ICD-10) codes: H00; H01; H02; H03; H04; H05; H06; H13; H15; H17; H18; H19; H20; H21; H22; H25; H26; H27; H28; H30; H31; H32; H33; H34; H35; H36; H40; H42; H43; H45; H46; H47; H48; H49; H50; H51; H52; H54; H55; H58; H59 [13].

Table S2. Average incidence of sick leaves per 1 000 WREI, accumulative average annual percent change, and cumulative percentage change in the incidence of all sick leaves over the study period (2008-2018).

	AI[1] per 1 000 sick leaves	95% CI of AI	AAPC (%)[2]	95% CI of AAPC (%)	CPC (%)[3]
**2008-2013**	165.2	161.9 to 168.4	1.1	-3.4 to 5.7	3.0
**2013-2018**	214.9	211.3 to 218.5	21.4*	3.0 to 43.0	88.6
**All period**	186.1	182.7 to 189.5	5.7*	2.7 to 8.8	94.3

1 Average Incidence per 1 000 labor absence; 2 Annual Average Percentage Change (%); 3 Cumulative Percentage Change (%)
